# Automated Text Analysis Based on Skip-Gram Model for Food Evaluation in Predicting Consumer Acceptance

**DOI:** 10.1155/2018/9293437

**Published:** 2018-01-22

**Authors:** Augustine Yongwhi Kim, Jin Gwan Ha, Hoduk Choi, Hyeonjoon Moon

**Affiliations:** ^1^Department of Food Science and Biotechnology, Sejong University, Seoul, Republic of Korea; ^2^Department of Computer Science and Engineering, Sejong University, Seoul, Republic of Korea

## Abstract

The purpose of this paper is to evaluate food taste, smell, and characteristics from consumers' online reviews. Several studies in food sensory evaluation have been presented for consumer acceptance. However, these studies need taste descriptive word lexicon, and they are not suitable for analyzing large number of evaluators to predict consumer acceptance. In this paper, an automated text analysis method for food evaluation is presented to analyze and compare recently introduced two jjampong ramen types (mixed seafood noodles). To avoid building a sensory word lexicon, consumers' reviews are collected from SNS. Then, by training word embedding model with acquired reviews, words in the large amount of review text are converted into vectors. Based on these words represented as vectors, inference is performed to evaluate taste and smell of two jjampong ramen types. Finally, the reliability and merits of the proposed food evaluation method are confirmed by a comparison with the results from an actual consumer preference taste evaluation.

## 1. Introduction

Numerous companies which are launching products are interested in consumer opinions about their released products. Because this feedback can be used for marketing and improvement in their launched products, many companies regard consumer's opinion as the most vital information. Generally, consumer survey is considered the best option to gather information and analyze the feedback of consumer on the products. However, survey for data collection and analysis of user's feedback have two major drawbacks: (1) significant costs in terms of amount of time and money and (2) limited sample space in terms of survey participants. Especially regarding the food, many consumers have very subjective opinions. Hence, the data acquired from the survey are difficult to give useful information that reflect consumer's acceptance or preference because of its low sample space. For these reasons, most of the food companies rely on food specialists' opinion when they release, improve, and inspect their foods.

Recently, it has been observed that people share their experience of all the aspects of life on social networking services. As various SNS become dominant, huge text data were generated by heterogeneous group of users on the Internet. In particular, the contents of these huge text data have a lot of food reviews. Therefore, collecting and analyzing these text reviews automatically can overcome the drawbacks of the survey. In conventional food sensory analysis, studies [1–3] have been conducted to analyze and evaluate taste or smell words of foods. However, these studies need sensory word dictionaries built manually to evaluate taste and smell of foods. In this article, an automated text analysis framework is presented that does not need any additional sensory lexicon for food evaluation. The proposed method used two natural language processing techniques, morpheme analysis and word embedding for text analysis.

For several years, as word representation methods for natural language processing techniques have been suggested [4, 5], we focused on the possibility of automated text analysis technique for users' review analysis. In particular, as deep-learning based approaches for word representation methods [5, 6] were proposed recently, it is possible to analyze large text data and reflect their meaning as vector more accurately, that is, converting words into vectors by their meaning, and it is then possible to compute the similarities between words and infer their meaning.

The goal of the proposed method is to automatically evaluate food products from the large number of reviews to measure consumer acceptance or preference. Based on word representation method, we evaluated two jjampong (mixed vegetables and seafoods soup) ramen types as introduced recently and relatively high end prized instant ramen types of taste, smell, and characteristics from online reviews. We also verify the taste evaluation result acquired from the proposed method by comparing descriptive sensory evaluation results about taste and smell of two jjampong ramen types acquired from approximately 40 people. Currently, the food evaluation did not use strength of big data relying only on manual surveys to gather and analyze the consumers' feedback. The two main contributions of this paper are (1) lowering the barriers to the application of big data and natural language processing techniques to food evaluation and (2) automatically analyzing the large number of text-based reviews for food evaluation to measure consumers' acceptance or preferences.

The organization of this paper is as follows. In Section 2, we illustrate related research on food sensory analysis and natural language processing.  Section 3 covers the proposed methodology; then, Section 4 provides details of experimental results. Finally, we discuss the effect of current work and draw conclusions.

## 2. Related Work

### 2.1. Big Data

Big data contains huge and diverse information, which can be used in various fields such as marketing, customer management, criminal investigations, politics, and economy [8]. In addition, there are various other fields where big data has shown significant contribution. However, very few researchers have applied big data in the food industry. According to research [9] of keywords analyzing related big data, it has been observed that traffic, health, disaster prevention, politics, economy, culture, and tourism fields mainly utilize big data, but food industry rarely utilizes big data. However, recent “Food Navigator” that can identify food habits and health of consumers through big data analysis was reported [10]. This can be seen as a good sign that the research is in progress to apply big data in the food industry.

### 2.2. Word Representation Methods

**Figure 1:**
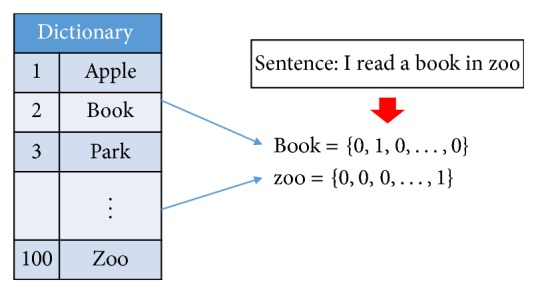
An example of one-hot representation method.

With the recent advances in deep-learning technology, text data analysis has seen significant development. According to the state-of-the-art researches, a variety of deep-learning based natural language processing (NLP) systems have been proposed such as translation system [11, 12] and speech recognition system [13]. In recent years, most NLP systems and technology cover research related to word embedding and its vector representation in order to use computer aided systems for semantic and syntactic analysis of text [5]. Word embedding represents words as numerical values; over the past years, various word representation methods were proposed [4]. Among these methods, one-hot representation is the simplest method to represent words as vectors.  Figure 1 illustrates the one-hot representation method, it represents words as vectors which are the same size as vocabulary dictionary. For example, if there are 100 words in a dictionary, words in a sentence represent 100-dimensional vector as shown in Figure 1. This representation method has helped to quantify the words so that they could be perceived by the computer. Also it has been used for junk mail classification [14] and widely as word features. However, one-hot representation has some disadvantages. One of the disadvantages of one-hot representation method is that the elements in vector space representation of a word are not meaningful. As a result, utility of these word vectors is mostly limited to the inference of words by computation such as similarity. Moreover, the larger the size of the dictionary is, the larger the length of the represented vectors.

In order to overcome these disadvantages, various word embedding models have been proposed to represent words in a low-dimensional space and reflect their meaning in a vector by applying feature learning techniques to word features acquired from one-hot representation. There are various techniques for dimensionality reduction such as principal component analysis, which performs a linear mapping of the data to a lower-dimensional space, where variance of data and its representation is maximized. Low-dimensional space converts a set of observations of possibly correlated variables into a set of values of linearly uncorrelated variables, reducing the redundancy in the data. In particular, word embedding models which are based on artificial neural network (ANN) and “Distributional Hypothesis” [15] showed high performance. Distributional Hypothesis means that “words with similar distributions have similar meanings” and helps to learn the meaning of the words.

Bengio et al. firstly designed feedforward neural network based language model (ff-NNLM) [16] for word representation and learned a distributed representation for words which greatly contributed to the improvement of the word representation. This approach inspires many researchers. As a result, ANN based language model was proposed [5]. Several years later, Mikolov and Tomas proposed recurrent neural network based language model (RNNLM) [17]. They modified ff-NNLM to recurrent neural network architecture and they improved the training speed and overcame the disadvantages of ff-NNLM such as fixed number of searching words problem. Recently, the state-of-the-art methods of word representation are word2vec models which were proposed by Mikolov et al. They proposed two models: (1) continuous bag-of-words (CBOW) model and (2) continuous skip-gram model. ANN based language model usually needs a billion or more words for training. These two models contributed to the reduction of the amount of computation, and these models were faster than previously proposed models such as ff-NNLM and RNNLM. Also they showed more improved performance; according to Mikolov's experimental results [5], skip-gram model has the highest accuracy in semantic word relationship test among RNNLM, NNLM, and CBOW.

### 2.3. Flavor Evaluation

One of the research areas of interest in food sensory analysis is flavor evaluation. So far, the studies [1–3] have focused on Korean sensory descriptive words for flavor profile evaluation. Therefore, these studies need sensory lexicon built manually. According to previous studies for building lexicon, they relied on questionnaire or collecting adjectives from the Internet manually. Based on the built sensory lexicon, they analyze the similarity and relationship between sensory words by statistical or mathematical method such as rough clustering [2] and fuzzy relation analysis [3]. In conclusion, previously proposed methods for the evaluation of flavor profile and qualitative sensory evaluation require the building of lexicon.

## 3. Proposed Framework

**Figure 2:**
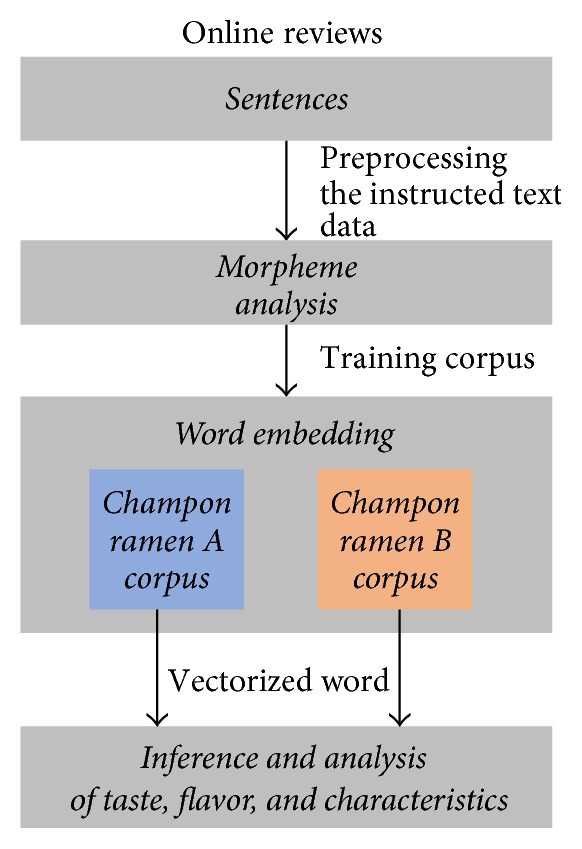
Overview of the proposed framework for automated text analysis in online food reviews.

The overview of the proposed automated text analysis method for online food reviews is shown in Figure 2. Users' reviews are categorized into two types of jjampong ramen (jjampong ramen A and jjampong ramen B) which are collected from SNS (https://section.blog.naver.com/). Based on these reviews, taste, smell, and characteristics of each jjampong ramen are analyzed. Firstly, words are extracted from review sentences by applying morpheme analysis; then these extracted words are used as each jjampong ramen corpus for training continuous skip-gram model. In morpheme analysis steps, we only consider nouns, adjectives, and verbs. Secondly, all words in each jjampong ramen corpus are represented as vectors. In this proposed framework, we infer and analyze represented word vectors to evaluate and compare two jjampong ramen types taste, smell, and characteristics in three ways: (1) word embedding analysis for estimating taste and smell; (2) clustering structure analysis for finding characteristics; (3) inference of the relationship between food taste and characteristics.

### 3.1. Data Acquisition

Users' reviews on two types of jjampong ramen are collected from SNS (https://section.blog.naver.com/). A total of 8999 reviews were collected with 4000 reviews for jjampong ramen A and 3999 reviews for jjampong ramen B, and from these reviews, 141,366 and 180,692 sentences were extracted from jjampong ramen A and jjampong ramen B, respectively.

### 3.2. Morpheme Analysis

In order to analyze acquired online reviews using the word embedding technique, an efficient corpus is required for the training of word embedding model. To build each jjampong ramen corpus, words were extracted from the collected sentences by morpheme analysis. This process is usually called preprocessing in text analysis. The Korean analysis package [18] was used for morpheme analysis and in this step, the useless morphemes were removed. Useless morphemes stand for postposition, special character, end of a word, and so forth, we extract only nouns, adjectives, and verbs. In addition, a stopword dictionary containing words unnecessary for analyzing such as “this,” “that,” and so on was constructed to remove meaningless words. The purpose of using not only nouns but also adjectives and verbs as training set for continuous skip-gram model is that the meaning of nouns can vary greatly depend on the adjectives and verbs used in the sentence due to the nature of the vocabulary.  Table 1 shows an example of word extraction from a sentence by morpheme analysis.

### 3.3. Word Embedding

Word embedding converts huge list of words extracted from text documents into vectors which have a low dimension, usually from 10 to 1000 dimensions. Text analysis generally ignores sentences, paragraphs, and order of the words and analyzes only the frequency of occurrence of words in the documents. However, this type of analysis may limit the understanding of words meaning in the sentence because the contextual meaning of the words and the appearance of the words are excluded from the analysis. Thus, to understand users' reviews more accurately, we applied word embedding technique to understand the structural characteristics and meaning of the words.

#### 3.3.1. Continuous Skip-Gram Model

**Figure 3:**
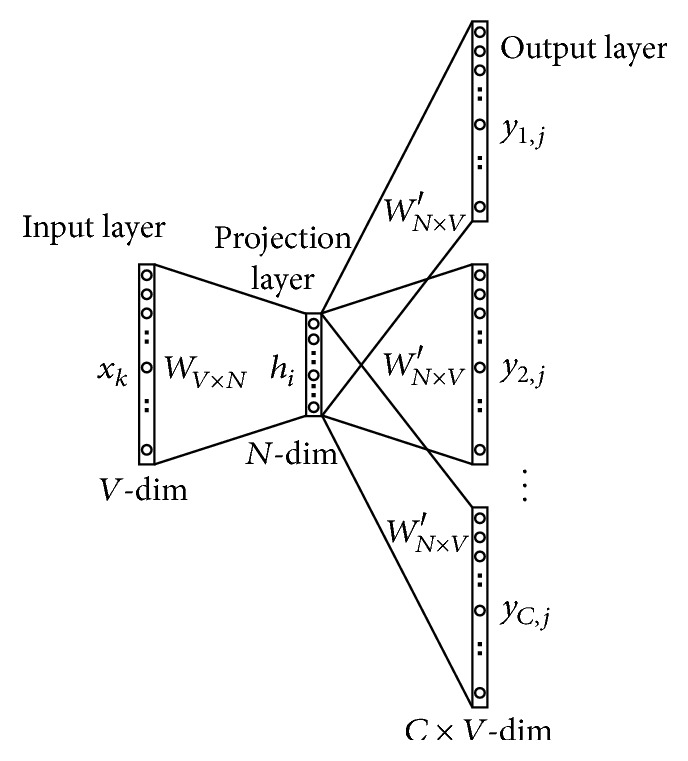
Continuous skip-gram model [7].

Tomas Mikolov developed word embedding model [5, 6] called continuous skip-gram model as shown in Figure 3. It consists of three layers: an input layer, projection layer, and hidden layer. Continuous skip-gram model predicts words that can appear in the neighborhood of the current word. Words used in the input layer are initialized by one-hot encoded vector, which means for a given input context word, only one out of *V* units, {*x*_1_, …, *x*_*V*_}, will be 1, and all other units are 0. Size of one-hot encoder vector is the same as the size of the vocabulary dictionary used in training. All the words used in the input layer are projected to an* N*-dimensional vector by a projection matrix *W* of *V* × *N* size and used as input to the projection layer. Output of the projection layer is multiplied by the weight matrix *W*′ of *N* × *V* size and fed to output layer. Finally, output layer predicts* C* adjacent words through softmax function. Predicted words are compared with the actual surrounding words of the input word, and the error is calculated, whereas the weight matrix *W*′ is updated so that the error rate is minimized. Therefore, if two words are used consistently in a similar context, the two words will have similar vector values, and various inferences and analysis can be made based on them.

#### 3.3.2. Training Model

Two skip-gram models are trained in the same process using each jjampong ramen corpus built by morpheme analysis, and words are represented as 64-dimensional vector. Four parameters are considered in training step. First is window size (this corresponds to *C* in Figure 3 and *C* = 4 in the underlying framework) that is the maximum distance between the current and predicted word within a sentence. Second parameter is minimum count: we ignore all words with total frequency lower than this in training step, and we select the minimum size as 10. Third and fourth parameters are number of iterations and learning rate. During training the proposed system, 500 iteration were made and learning rate linearly dropped from 0.025 to 0.001. Skip-gram model predicts words as softmax function in output layer. However, softmax function is computationally expensive; it takes much time for training. So, we used hierarchical softmax [6, 7] instead of softmax function in output layer to reduce time complexity. Time complexity of the skip-gram model using softmax is as follows:

  time for projecting the current word: *N*,
  time for computing the output: *N* × *V*,
  maximum distance between the current and predicted word: *C*,
  time complexity: *C*(*N* + *N* × *V*).

**Figure 4:**
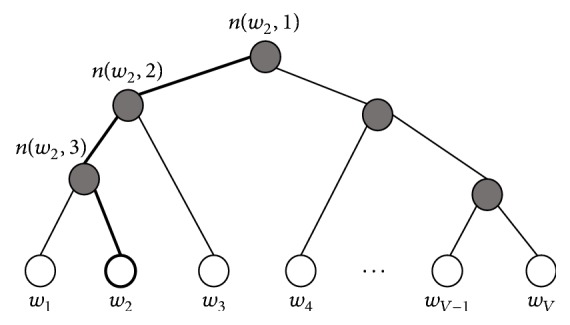
Example of hierarchical softmax.

For hierarchical softmax, as shown in Figure 4, we used a binary tree to calculate the probability and predict words. In Figure 4 white circles represent words in a corpus and *n*(*w*, *j*) means the *j*-th unit in the path from the root to the word *w*. Therefore, hierarchical softmax predicts words by the path from root to the leaf by multiplying the probability. Unlike softmax function, hierarchical softmax does not need to search whole words, so we can reduce the computation time in output layer to *N* × log_2_*V*. Thus, time complexity of the skip-gram model using hierarchical softmax is as follows:

  enhanced time complexity: *C*(*N* + *N* × log_2_*V*).

Hence, we could train skip-gram model much faster than the previously proposed word embedding models such as ff-NNLM [16] and RNNLM [17]. In addition, applying hierarchical softmax in the output layer, we do not use weight matrix *W*′ of existing skip-gram model. We use *V* − 1 internal nodes; each has a weight vector of size *N* called *v*′*i* and is updated according to errors which occur during the training process instead. Through the above-mentioned process, we trained two skip-gram models with each jjampong ramen corpus. Both of these skip-gram modles are analyzed on users reviewers collected from different blogs.

### 3.4. Inference for Food Evaluation

#### 3.4.1. Main Taste and Smell Estimation

We are interested in the taste and smell of foods; most of the users share their experiences on the online reviews. Therefore, to find main taste and smell in online reviews, we select “*Mat* (taste)” and “*Hyang* (smell)” words as keywords and represent them as vectors through trained skip-gram model and compute similarity with words included in each jjampong ramen corpus. Hence, if words in corpus have high similarity with “*Mat* (taste)” or “*Hyang* (smell),” that means specific taste or smell of two jjampong ramen types is highly shared by users in reviews.  Algorithm 1 shows the algorithm for main taste and smell estimation. Similarity between words is computed by cosine similarity which is defined as

(1) $$\begin{array}{c} \text{cosine similarity}=\frac{\sum_{i=1}^{n}A_{i}\times B_{i}}{\sqrt{\sum_{i=1}^{n}{\left(A_{i}\right)}^{2}}\times \sqrt{\sum_{i=1}^{n}{\left(B_{i}\right)}^{2}}}, \end{array}$$

where *A*, *B* are converted word vectors and *n* is set to 64.

The algorithm for jjampong ramen taste and smell estimation, specified in Algorithm 1, has three parameters: keyword, corpus, and number of words in corpus. Only “*Mat* (taste)” or “*Hyang* (smell)” is used as keyword. Then, the keyword is represented a vector, and similarity between the keyword and all of words in corpus is computed. By extracting words which have top 20 high similarities with keywords and removing noise words, we estimate jjampong ramen taste and smell. The “filtering” method in the algorithm means removing noise words, that is words unrelated to taste or smell such as “*Na* (I),” “*Jin-jja* (real),” and “*Teug-yu* (unique).”

Tables 2 and 3 show the smell and evaluation result of each jjampong ramen. Smell and taste words are extracted: 11 and 8 words, respectively. It can be seen that overall similarity of taste words is much higher than similarity of smell words. It means that majority of users highly mentioned the taste rather than smell and are more interested in taste than smell. In addition, the estimated taste and smell words in the two jjampong ramen types are the same, but they have variations in similarity scores.

For Korean, the taste related descriptive language was the sensory stimulations in mouth that might be related to taste and retronasal sensation. The flavor descriptive language was usually based on smell before eating. However, in Korean language flavor descriptors are used interchangeably.

**Figure 5:**
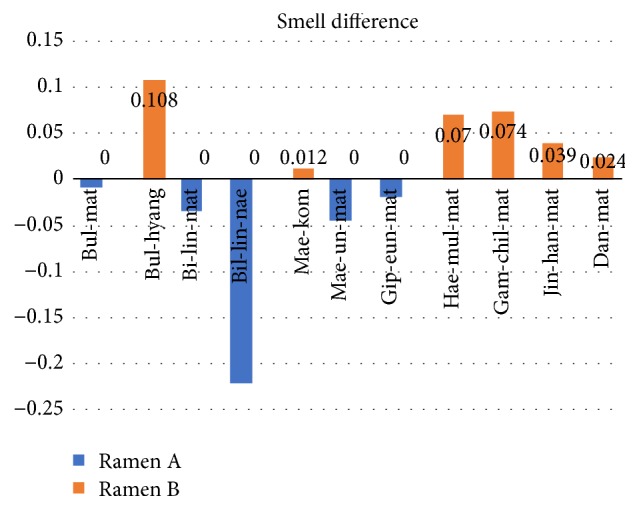
Difference in smell between two jjampong ramen types.

**Figure 6:**
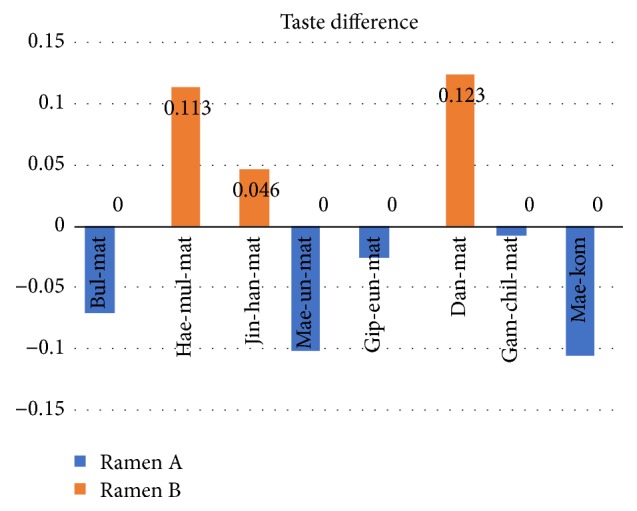
Difference in taste between two jjampong ramen types.

If the word has a gap in similarity between two jjampong ramen types, it could be representative smell or taste of each jjampong ramen. Figures 5 and 6 show the difference between smell and taste words' similarities of two jjampong ramen types. The positive value (red bar) means that jjampong ramen A has more strong smell or taste; the negative value (blue bar) means that jjampong ramen B has more strong smell or taste.

Figure 5 shows difference in smell of each jjampong ramen, “*Bul-hyang* (burning vegetables flavor cooked with wok),” “*Gam-chil-mat* (rich flavor),” and “*Hae-mul-mat* (seafood flavor)” mean strong smell in jjampong ramen A, and “*Bi-lin-nae* (off-fishy smell)” means strong smell in jjampong ramen B. And, as shown in Figure 6, “*Hae-mul-mat* (seafood flavor)” and “*Dan-mat* (sweetness)” mean more strong taste in jjampong ramen A, and “*Bul-mat* (wok flavor),” “*Mae-un-mat* (spicy with longer duration),” and “*Mae-kom* (spicy with short duration)” mean more strong taste in jjampong ramen B.

#### 3.4.2. Clustering Structure Analysis

In order to find the characteristics of two jjampong ramen types, we analyzed clustering structure by projecting words in a two-dimensional space. Therefore, frequency of noun words in reviews was calculated for projecting two-dimensional space; we find characteristics of two jjampong ramen types through the clustering structure analysis.

Table 4 shows the result of noun frequency from each jjampong ramen reviews and it was confirmed that “*Seu-peu* (powdery soup sauces),” “*Myeon* (noodle),” and “*Gug-mul* (soup)” are most frequent words in order of frequency in both jjampong ramen's reviews. However, these three words are typical ramen characteristics; they cannot become of each jjampong ramen characteristic, whereas the other frequency words belong to typical ramen characteristics (“*Seu-peu* (powdery soup sauces),” “*Myeon* (noodle),” and “*Gug-mul* (soup)”); namely, that means properties of typical ramen characteristics. Hence, we identify relationship of properties and typical ramen characteristics by projecting words in a two-dimensional space.

In order to project words in a two-dimensional map, we used t-SNE [19] technique proposed by van der Matten and Hinton which is based on initial studies on SNE [20] and used for multidimensional scaling. In t-SNE method, input high dimensional data *X* = {*x*_1_, *x*_2_, *x*_3_,…, *x*_*n*−1_, *x*_*n*_} is first converted into a low-dimensional data space *Y* = {*y*_1_, *y*_2_, *y*_3_,…, *y*_*n*−1_, *y*_*n*_}. The specific process of converting high-dimensional point to low-dimensional data through the t-SNE method is as follows. Priority and distance are converted to joint probability distribution *P*, all of pairwise of high-dimensional data, and the matrix *P* is defined as in (2). Then, joint probability *q*_*ij*_ is defined as in (3); it means similarity between *y*_*i*_ and *y*_*k*_ which corresponds to *p*_*ij*_:

(2) pij=exp−δ2ij/σ∑k∑l−kexp−δ2ij/σ,pij=0

(3) qij=1+yk−yi2−1∑k∑l−k1+yk−yi2−1,qij=0.

As with *p*_*ij*_, *q*_*ij*_ is also defined as zero. Finally, the error between the two distributions *P* and *Q* is calculated through Kullback-Leibler divergence. The error is minimized through gradient descent method and the cost function *C*(*Y*) is defined as

(4) $$\begin{array}{c} C\left(\;Y\;\right)=KL\left(\;P||Q\;\right)=\sum_{i}\sum_{i\neq i}p_{iij}log\frac{p_{ij}}{q_{ij}}. \end{array}$$

Table 5 shows the words contained in each cluster and distinct words between two jjampong ramen types are marked as bold and underlined. Overall, two jjampong ramen types have similar properties regarding three typical ramen characteristics; however properties contained in each typical ramen show slight difference. Specifically, we can infer that jjampong ramen A has more chewy texture; on the other hand, jjampong ramen B has flavor of wok in “*Myeon* (noodle).” We can also infer that “*Gug-mul* (soup)” of jjampong ramen A is based on various vegetables; on the other hand, “*Gug-mul* (soup)” of jjampong ramen B is based on vegetables and meat.

**Figure 7:**
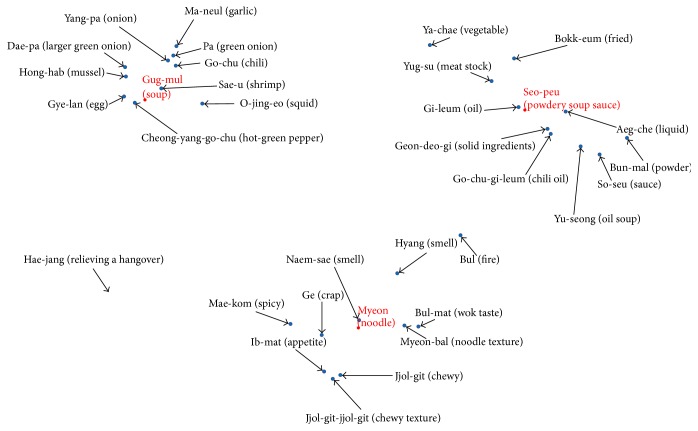
Cluster structure of correlations between typical characteristics and noun words in jjampong ramen A.

**Figure 8:**
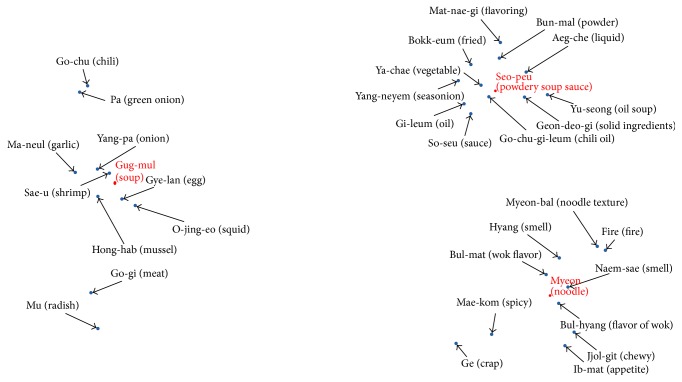
Cluster structure of correlations between typical characteristics and noun words in jjampong ramen B.

Through this process, words are projected into a two-dimensional space. Figures 7 and 8 show the results of visualized clustering structure of two jjampong ramen types. It can be seen that the closer the distance between words is, the more similar they are. As a result of visualization, jjampong ramen reviews have three-cluster structure with typical ramen characteristics (“*Seu-peu* (powdery soup sauce),” “*Myeon* (noodle),” and “*Gug-mul* (soup)”).

#### 3.4.3. Relationship Analysis between Taste and Characteristics

Through the above process, we found the food taste, smell, and characteristics from users' reviews. The most vital information in food would be the taste. Therefore, we analyze the relationship between taste and typical ramen to infer the characteristics that have the greatest effect on taste. In order to infer relationship between taste and characteristics, we created representation vector of each cluster's words and calculated similarities between three representation vectors and 8 taste words as shown in Table 2. Representation vectors of each cluster (“*Seu-peu* (powdery soup sauce),” “*Myeon* (noodle),” and “*Gug-mul* (soup)”) are as follows:

(5) $$\begin{array}{c} \text{representation vector}=\frac{\sum_{i}^{n}w_{i}}{n}, \end{array}$$

where *n* is the number of words in cluster and *W* is the word vectors in cluster.

Since representation vector is based on cluster words and cluster words are also based on the frequency of noun words mentioned by users, it can effectively represent users' opinion about typical ramen characteristics. By computing similarities between representation vector and taste words, how characteristics concern with taste of each jjampong ramen can be grasped.  Algorithm 2 shows the algorithm for inference of relationship between cluster representation vectors and taste words.  Figure 8 shows the similarities between cluster words and taste words visualized as perception map.

**Figure 9:**
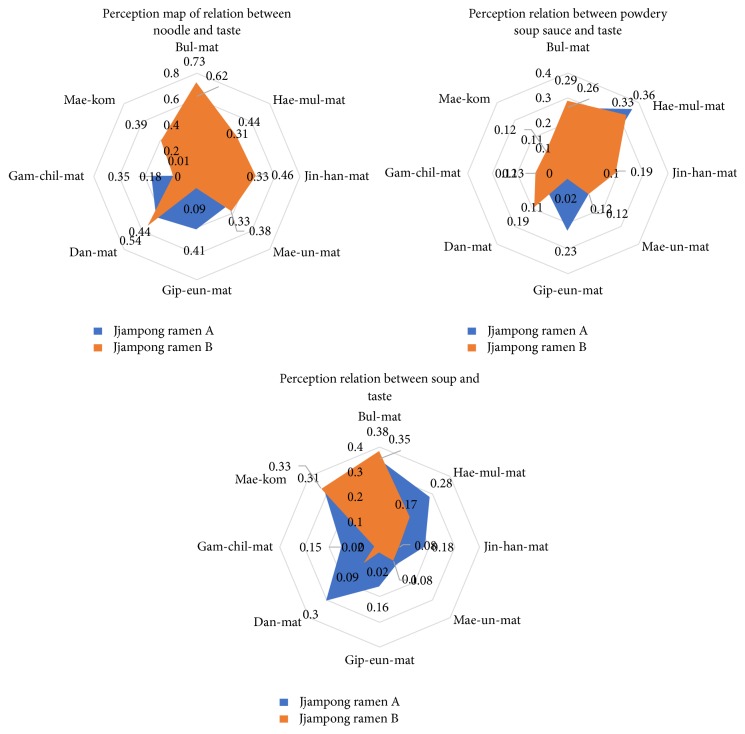
Perception maps of relation between typical characteristics and taste.

In Figure 9, it can be seen that the larger the area occupied by perception map is, the higher the relationship with taste words is. Among them, relationship of “*Myeon* (noodle)” and taste occupied extensive area. In other words, “*Myeon* (noodle)” is the most related to taste and majority of consumers are interested in taste of noodle. Also each jjampong ramen shows differences of taste in three typical ramen characteristics. The most interesting point in Figure 8 is the similarity between “*Gug-mul* (soup)” and taste. Even though it does not occupy much area of perception map, it shows that jjampong ramen A has a balanced taste, but jjampong ramen B is prone to taste of “*Bul-mat* (wok flavor)” and “*Mae-kom* (spicy with short duration)” taste in “*Gug-mul* (soup).” On the other hand, most consumers do not recognize much difference between “*Seu-peu* (powdery soup sauce)” and taste of two jjampong ramen types.

## 4. Experiment Result

In this paper, we analyzed two jjampong ramen types reviews collected from SNS to evaluate taste, smell, and characteristics. By training continuous skip-gram model for word representation as a vector, we analyzed reviews in three ways based on represented word vector for food evaluation. At first, as a result of taste and smell evaluation, two jjampong ramen types have 8 kinds of tastes. Specifically, jjampong ramen A has strong “*Hae-mul-mat* (seafood flavor)” and “*Dan-mat* (sweetness)”; jjampong ramen B has strong “*Bul-mat* (wok flavor),” “*Mae-un-mat* (spicy with longer duration),” and “*Mae-kom* (spicy with short duration)” in taste. Also two jjampong ramen types have 11 kinds of smell: jjampong ramen A has strong “*Bul-mat* (burnt),” “*Hae-mul-mat* (seafood flavor),” and “*Gam-chil-mat* (rich flavor)”; jjampong ramen B has strong “*Bi-lin-nae* (off-fishy smell)” in smell.

Secondly, we analyzed jjampong ramen characteristics by computing noun words frequency and projecting them in a two-dimensional space. Jjampong ramen has three typical characteristics such as “*Seu-peu* (powdery soup sauce),” “*Myeon* (noodle),” and “*Gug-mul* (soup)”; we analyzed clustering structure based on these typically characteristics. As a result of clustering analysis, differences exist in “*Myeon* (noodle)” and “*Gug-mul* (soup)” between two jjampong ramen types. Based on these results, we inferred that jjampong ramen has more “*Jjol-git-jjol-git* (enhanced chewy texture)” in “*Myeon* (noodle)” and “*Gug-mul* (soup)” of jjampong ramen A based on various vegetables and “*Gug-mul* (soup)” of jjampong ramen B based on meat.

Lastly, we inferred characteristics which affect taste more by analyzing relationship between taste and typical ramen characteristics. As a result of inference, “*Myeon* (noodle)” has the most significant influence on taste and jjampong ramen A has more balanced taste in “*Gug-mul* (soup)” than jjampong ramen B.

To verify the reliability of these results, we conducted a taste survey from 40 users who have experienced two jjampong ramen types. The survey has been proceeded to write more than five taste of flavor expressions related to each jjampong ramen type per user.  Table 6 shows the survey results. Although the samples of the questionnaire are too small to fully generalize the taste expressions of two jjampong ramen types, the results of questionnaire correspond to the results of the method which is proposed in this paper. More specifically, according to questionnaire results,* Hae-mul-mat* (seafood flavor) is strong taste in jjampong ramen A; meanwhile,* Bul-mat* (wok flavor) and* Mae-un-mat* (spicy with short duration) are more strong tastes in jjampong ramen B. Also* Jjol-git-ham* (chewy) has more frequency in champon ramen A; it means that jjampong ramen A has more chewy texture than jjampong ramen B. When comparing these questionnaire results and the results derived by the automated text analysis method proposed in this paper, we find that they are the same results in terms of taste and characteristics. Through this comparison, we verified reliability of taste and characteristics inference result derived by proposed method in this paper. Moreover, this proposed automated text analysis method can provide not only simple taste information but also various and detailed information regarding the food.

## 5. Conclusion

In this study, we proposed automated text analysis method for two jjampong ramen types evaluation by analyzing text reviews acquired from SNS. We analyzed text reviews through representing words by vectors, and based on these vectors we inferred taste, smell, and characteristics of two jjampong ramen types. Moreover, we compared the results of the questionnaire conducted by approximately 40 people and verified the reliability of inference results.

For several years, many studies have been conducted for sensory analysis in food sensory evaluation for measuring consumer acceptances or preference. However, these studies usually need a manually built sensory lexicon. Since they relied on questionnaire or manual work, there were a lot of efforts involved in building the lexicon.

In this paper, we tried to overcome these difficulties of previous work by collecting users' food reviews in SNS and analyzing acquired text data through word representation method. This study gives three advantages: (1) a sensory word lexicon is not required; (2) by using a large amount of users' opinion, this study can provide more detailed and generalized food evaluation result; (3) this result can be widely utilized not only for evaluating food but also for marketing or improving products at food market.

In this study, we only considered taste and smell out of five senses. However, sight and touch are also important and sensory for food evaluation as well. Therefore, this paper is room for improvement and we can evaluate the food in in more detail by improving this paper.

Food industry has not utilized big data or natural language processing methods in research so far. So through this paper, we expect that the convergence research of computer engineering and food science will actively be continued.

## Figures and Tables

**Algorithm 1:**
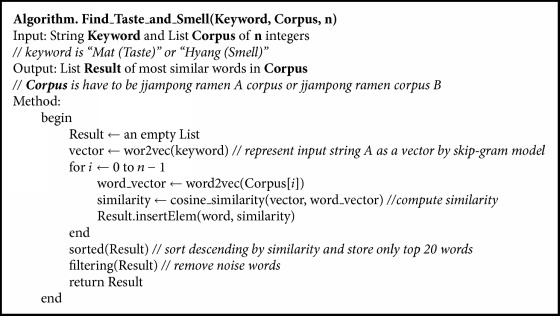
Algorithm for taste and smell analysis.

**Algorithm 2:**
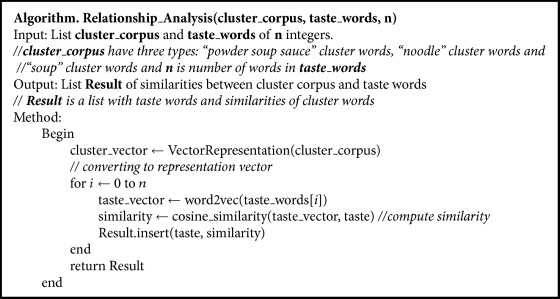
Algorithm for relationship analysis.

**Table 1:** Example of word extraction from a sentence by morpheme analysis.

Sentence: “I ate rich seafood taste and soup taste jjampong”	
Words	Morpheme
Soup taste	Noun
Seafood taste	Noun
Rich	Adjective
Jjampong	Noun
Ate	Verb

**Table 2:** Smell evaluation result of each jjampong ramen type.

Words	Flavor description	Similarity of jjampong ramen A	Similarity of jjampong ramen B
*Bul-mat*	Burnt	0.69	0.7
*Bul-hyang*	Burning vegetables flavor cooked with wok	0.67	0.56
*Bi-lin-mat*	Off-fishy flavor	0.37	0.41
*Bi-lin-nae*	Off-fishy smell	0.40	0.62
*Mae-kom*	Spicy smell with short duration	0.38	0.37
*Mae-un-mat*	Extended spicy smell	0.36	0.40
*Gip-eun-mat*	Mixed (balanced) flavor	0.40	0.42
*Hae-mul-mat*	Sea food flavor	0.50	0.43
*Gam-chil-mat*	Rich flavor (umami)	0.46	0.38
*Jin-han-mat*	Complexed rich flavor	0.42	0.38
*Dan-mat*	Sweet flavor	0.41	0.39

**Table 3:** Taste estimation result of each jjampong ramen type.

Words	Flavor description	Similarity of jjampong ramen A	Similarity of jjampong ramen B
*Bul-mat*	Wok flavor	0.84	0.91
*Hae-mul-mat*	Sea food flavor	0.75	0.63
*Jin-han-mat*	Complexed rich flavor (without being distinguishable)	0.54	0.49
*Mae-un-mat*	Spicy with longer duration	0.52	0.62
*Gip-eun-mat*	Kokumi-like	0.52	0.55
*Dan-mat*	Sweetness	0.65	0.52
*Gam-chil-mat*	Umami	0.48	0.49
*Mae-kom*	Spicy with short duration	0.48	0.58

**Table 4:** Frequency of noun words that can be used in noticeable characterization in consumer preference of each jjampong ramen type.

Words	Description	Freq.
*jjampong ramen A*		
*Seu-peu*	Powdery soup sauce	11761
*Myeon*	Noodle	5974
*Gug-mul*	Soup	4331
*Geon-deo-gi*	Solid ingredients	3694
*Yu-seong*	Oily	3014
*Myeon-bal*	Noodle texture	2797
*Aeg-che*	Liquid	2532
*Bul*	Fire/spicy	2127
*Bul-mat*	Wok taste	1385
*So-seu*	Liquid sauce	920
*Ib-mat*	Appetite	852
*Hyang*	Smell	848
*O-jing-eo*	Squid	820
*Gye-lan*	Egg	742
*Ya-chae*	Vegetable	647
*Mae-kom*	Spicy	639
*Gi-leum*	Oil	614
*Naem-sae*	Smell	585
*Pa*	Green onion	583
*Ge*	Crap	583
*Bokk-eum*	Fried	563
*Jjol-git*	Chewy	526
*Go-chu-gi-leum*	Chili oil	514
*Yang-pa*	Onion	492
*Bun-mal*	Powder	405
*Sae-u*	Shrimp	376
*Hong-hab*	Mussel	345
*Hae-jang*	Relieving a hangover	341
*Yug-su*	Meat stock	312
*Ma-neul*	Garlic	308
*Cheong-yang-go-chu*	Hot-green pepper	305
*Go-chu*	Chili	295
*Dae-pa*	Larger green onion	281
*Jjol-git-jjol-git*	Enhanced chewy texture	251

*Jjampong ramen B*		
*Seu-peu*	Powdery soup sauce	6978
*Myeon*	Noodle	5609
*Gug-mul*	Soup	4159
*Bul*	Fire/spicy	3695
*Myeon-bal*	Noodle texture	2794
*Geon-deo-gi*	Solid ingredients	2646
*Ya-chae*	Vegetable	2434
*Bul-mat*	Taste of fire	2034
*Bokk-eum*	Fried	1687
*Hyang*	Smell	1155
*Ib-mat*	Appetite	1005
*Mat-nae-gi*	Flavoring	891
*O-jing-eo*	Squid	779
*So-seu*	Liquid sauce	706
*Gi-leum*	Oil	695
*Mae-kom*	Spicy	689
*Gye-lan*	Egg	689
*Ge*	Crap	678
*Yu-seong*	Oily	678
*Pa*	Green onion	619
*Naem-sae*	Smell	612
*Go-gi*	Meat	592
*Bun-mal*	Powder	589
*Jjol-git*	Chewy	527
*Aeg-che*	Liquid	491
*Sae-u*	Shrimp	453
*Go-chu-gi-leum*	Chili oil	397
*Yang-pa*	Onion	336
*Yang-neyom*	Seasoning	334
*Bul-hyang*	Wok flavor	328
*Ma-neul*	Garlic	321
*Mu*	Radish	306
*Hong-hab*	Mussel	302
*Go-chu*	Chili	293

**Table 5:** Words which belong to each cluster (typical ramen characteristics).

Cluster	Jjampong ramen A		Jjampong ramen B	
	Words	Description	Words	Description
*Seu-peu *(soup sauce)	*Ya-chae*	Vegetable	*Ya-chae*	Vegetable
	*Bokk-eum*	Fried	*Bokk-eum*	Fried
	*Gi-leum*	Oil	*Gi-leum*	Oil
	*Aeg-che*	Liquid	*Aeg-che*	Liquid
	*Geon-deo-gi*	Solid ingredients	*Geon-deo-gi*	Solid ingredients
	*Go-chu-gi-leum*	Chili oil	*Go-chu-gi-leum*	Chili oil
	*Yu-seong*	Oil soup	*Yu-seong*	Oil soup
	*Bun-mal*	Powder	*Bun-mal*	Powder
	*So-seu*	Sauce	*So-seu*	Sauce
	***Yug-su***	**Meat stock**	***Mat-nae-gi***	**Flavoring**
	*-*	*-*	***Yang-neyom***	**Seasoning**

*Myeon *(noodle)	*Bul*	Fire	*Bul*	Fire
	*Hyang*	Smell	*Hyang*	Smell
	*Bul-mat*	Wok taste	*Bul-mat*	Wok taste
	*Myeon-bal*	Noodle texture	*Myeon-bal*	Noodle texture
	*Naem-sae*	Smell	*Naem-sae*	Smell
	*Ge*	Crap	*Ge*	Crap
	*Mae-kom*	Spicy	*Mae-kom*	Spicy
	*Ib-mat*	Appetite	*Ib-mat*	Appetite
	*Jjol-git*	Chewy	*Jjol-git*	Chewy
	***Jjol-git-jjol-git***	**Chewy texture**	***Bul-hyang***	**Wok flavor**

*Gug-mul* (soup)	*Hong-hab*	Mussel	*Hong-hab*	Mussel
	*Gye-lan*	Egg	*Gye-lan*	Egg
	*Ma-neul*	Garlic	*Ma-neul*	Garlic
	*Pa*	Green onion	*Pa*	Green onion
	*Yang-pa*	Onion	*Yang-pa*	Onion
	*Sae-u*	Shrimp	*Sae-u*	Shrimp
	*O-jing-eo*	Squid	*O-jing-eo*	Squid
	*Go-chu*	Chili	*Go-chu*	Chili
	*Cheong-yang-go-chu*	Hot-green pepper	*Cheong-yang-go-chu*	Hot-green pepper
	***Dae-pa***	**Larger green onion**	**Go-gi**	**Meat**
	***Hae-jang***	**Relieving a hangover**	**Mu**	**Radish**

**Table 6:** Top five taste expressions of two jjampong ramen types in questionnaire.

Words	Description	Freq.
*Jjampong ramen A*		
*Hae-mul-mat*	Sea food flavor	27
*Jjol-git-ham*	Chewy	24
*Geon-deo-gi-seu-peu*	Ingredient soup minced	11
*Kal-gug-su-myeon*	Handmade noodles	9
*Bul-hyang*	Burning vegetables flavor with wok	9

*Jjampong ramen B*		
*Bul-mat*	Wok taste	22
*Mae-un-mat*	Spicy	19
*Jjol-git-ham*	Chewy	17
*Pung-mi-yu-hyang*	Smell of flavored oil	9
*Myeon-ui-sig-gam*	Texture of noodles	8
